# The Use of an Advanced Intelligent–Responsive Polymer for the Study of Dynamic Water–Carbon Dioxide Alternating Displacement

**DOI:** 10.3390/polym16081040

**Published:** 2024-04-10

**Authors:** Feng Zhang, Jingong Zhang, Yidong Yuan, Zishu Yong, Zhuoyue Yan, Jiayuan Zhang, Guochao Lu

**Affiliations:** 1 Department of Geology, Northwest University, Xi’an 710069, China202010341@stumail.nwu.edu.cn (Z.Y.); 2 College of Geosciences, China University of Petroleum, Beijing 102249, China; 3 National Key Laboratory of Continental Shale Oil, Northeast Petroleum University, Daqing 163000, China; 4 College of Geosciences, Jilin University, Changchun 130061, China

**Keywords:** intelligent polymer, CO_2_ response, alternating displacement, plugging performance

## Abstract

Addressing the issue of inadequate temperature tolerance in traditional polymers, in this study, we successfully executed a one-step synthesis of intelligent–responsive polymers which have excellent adaptability in water–gas alternating displacement scenarios. Utilizing the fatty acid method, we produced OANND from oleic acid (OA) and N,N-dimethyl-1,3-propanediamine (NND). Upon testing the average particle size in the aqueous solution both prior and subsequent to CO_2_ passage, it became evident that OANND assumes the form of a small-molecule particle in the aqueous phase, minimizing damage during formation. Notably, upon CO_2_ exposure, it promptly organizes into stable micelles with an average size of 88 nm and a relatively uniform particle distribution. This unique characteristic endows it with a rapid CO_2_ response mechanism and the ability to form a highly resilient gel. In the exploration of viscoelastic fluids, we observed the remarkable behavior of the AONND aqueous solution when CO_2_/N_2_ was introduced. This system displayed repeatable transitions between aqueous and gel states, with the highest viscosity peaking at approximately 3895 mPa·s, highlighting its viscosity reversibility and reusability properties. The rheological property results that we obtained indicate that an elongated micellar structure is present in the solution system, with the optimal concentration ratio for its formation determined as 0.8, which is the molar ratio of the OANND-NaOA system. In the sealing performance tests, a 1.0 wt% concentration of the gel system exhibited excellent injectability properties. At 80 °C, this gel effectively reduced the permeability of a sand-filled model to 94.5% of its initial value, effectively sealing potential leakage paths or gas fluxes. This remarkable ability to block leakage paths and reduce seepage capacity highlights the material’s superior blocking effect and erosion resistance properties. Furthermore, even at a temperature of 90 °C and an injection pore volume (PV) of 3, this plugging system could reduce the permeability of a high-permeability sand-filled model to over 90% of its initial value.

## 1. Introduction

As the global energy consumption rate continues to rise, the impact of increased oil production on the world economy is becoming increasingly significant. However, following the conventional oil extraction methods used for crude oil, 65% of the product remains in the reservoir. Moreover, in recent years, it has been determined that the proportion of newly added oil resources in the world that are difficult to extract is close to 75% [1,2]. A reservoir’s pore throat structure is highly complex [3], resulting in poor physical properties and rapid production decline, further diminishing oil recovery efficiency. Among the various enhanced oil recovery techniques utilized in the field, the CO_2_ flooding technique employs multiple mechanisms [4,5], including viscosity reduction, diffusion, and miscibility, making it an effective extraction method. It has a wide range of applications, particularly in low-permeability and ultra-low-permeability reservoirs [6,7]. Additionally, it effectively reduces CO_2_ greenhouse gas emissions and promotes geological carbon storage [8]. However, due to the non-homogeneity of reservoir oil, CO_2_ injected during the CO_2_ drive stage tends to flow along high-permeability layers [9,10,11,12]. This results in the presence of a significant amount of unrecoverable crude oil in low-permeability layers, often leading to the gas flurry phenomenon [13,14,15]. This limits the efficiency of gas injection, resulting in poor oil drive performance and reduced production. Various methods have been proposed in the field to mitigate the occurrence of gas migration, among which the water alternating gas (WAG) process has attracted the attention of researchers [16,17].

CO_2_-responsive gels [18] can effectively reduce the mobility of CO_2_ and slow down the occurrence of gas channeling by reducing the relative permeability of CO_2_ during the CO_2_ flooding technique. Under the influence of gravitational differentiation, the water drive sweeps through the lower level of oil, an area that cannot be reached by the gas drive, further expanding the reach volume. By utilizing both gas and liquid phases to alternately sweep different oil-bearing pore channels [19], the macroscopic reach efficiency and recovery rate are improved. However, the gravitational bias and high mobility of the water limit its effectiveness [12] in reservoirs with a high-permeability ratio and thick oil layers. At present, researchers are utilizing polymers in water segment plugs to control the consistency of water or using polymer gels to seal large channels [17]. These approaches have been observed to improve oil-water flow ratios and enhance ripple efficiency outcomes [20,21]. The key to the effective application of this technology in the field lies in the high viscosity created by polymers [22]. However, conventional polymers suffer from poor temperature resistance properties in high-temperature subsurface environments. Thermal thinning leads to a decrease in viscosity, which compromises the polymer’s ability to recover effectively. While increasing a polymer’s molecular weight can mitigate viscosity loss, it also results in a higher initial viscosity of the polymer solution, leading to increased injection pressure; the solution will also suffer from damage and have a worse sealing performance [23,24] for CO_2_-channeling and -control purposes. Developing a novel water-CO_2_ intelligent flow control system with superior temperature resistance properties remains a significant challenge in the literature.

The research objective of this study is to improve the effectiveness of the CO_2_ and water combined displacement process by developing CO_2_-responsive polymers to enhance the sweep efficiency of the displacement system. Based on the intelligent properties [25,26,27] of CO_2_-responsive materials, the CO_2_ intelligent-responsive surfactant oleamide propyl tertiary amine, which has a good response effect and is easy to bind with counterions, is selected as the stimulus object. By introducing organic salt counterions, we create a cation-based-response micellar system using a counterion-induced action. Furthermore, we innovatively designed an intelligent-responsive polymer that functions as a mobility control agent for the CO_2_ channel. This polymer is driven by water-carbon dioxide alternation and effectively scavenges gases. The system exhibits low viscosity results prior to CO_2_ response, facilitating improved injection of the product into deeper layers. The CO_2_ triggers a rapid response, resulting in the formation of a high-strength gel. Concurrently, a series of plugging experiments are designed to elucidate the system’s mechanism of action, and its blocking performance is also evaluated. These results provide innovative ideas for oilfield gas tamper control techniques.

## 2. Experimental Methods

### 2.1. Experimental Materials and Synthesis Methods

The following reagents were used in this study: oleic acid (OA, AR, Aladdin, Shanghai, China), N,N-dimethyl-1,3-propanediamine (NND, AR, Aladdin, Shanghai, China), sodium oleate (NaOA, AR, Aladdin, Shanghai, China), sodium p-toluenesulfonate (SPTS, AR, Aladdin, Shanghai, China), CO_2_ (>99.99%, Chengdu Jinli Gas Company, Chengdu, China), and N_2_ (>99.99%, Chengdu Jinli Gas Company).

The crude oil sample that we used was provided by a Chinese oilfield, and the artificial cubic cores were purchased from Beijing Dongfang Zhisheng Petroleum Technology Co., Ltd. (Beijing, China). All other consumables and chemical reagents were obtained from commercial sources.

The experimental apparatus used in our study is presented in Table 1.

### 2.2. Synthesis and Preparation of Polymers

**Figure 1:**
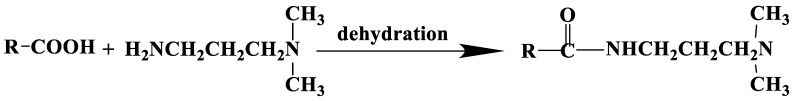
Synthesis route of polymers’ characterization and performance evaluation.

We used the fatty acid method in our study. We slowly added 45 mL of N,N-dimethyl-1,3-propanediamine (NND) to a 250 mL three-neck flask by using the condensation reflux technique, a water separator, a thermometer, and a stirring device with 100 mL of oleic acid (OA), under the protection of nitrogen with a constant-pressure dropping funnel; the dropping process was completed to increase the temperature in the flask to 155 °C. The reaction was then refluxed at a constant temperature with the magnetic stirring technique for about 10 h to obtain a yellow oily liquid. Subsequently, the yellow liquid that we created was distilled under a reduced-pressure condition for 0.5 h while it was still hot, and the water and excess N,N-dimethyl-1,3-propanediamine were withdrawn and cooled down to obtain the final product of water-CO_2_ alternate gas to control the scavenging capabilities of the intelligent-responsive polymers. The synthetic roadmap used for this process is presented in Figure 1.

#### 2.2.1. Infrared Spectroscopy

The Fourier transform infrared spectroscopy KBr method [28] was used to test the samples and determine their characteristic functional groups. The main role of infrared spectroscopy is to analyze the chemical composition of substances; it is based on the specific absorption peak position and shape of special groups to infer the structure of material groups according to the strength of the characteristic peaks and to determine the content of each component in the mixture. In addition, it has several positive characteristics, including a rapid performance time and high sensitivity, it requires a small amount of specimen, and it can effectively analyze solid-liquid specimens. Therefore, infrared spectroscopy has become one of the most commonly used methods for performing material characterizations.

#### 2.2.2. ^1^H Magnetic Nuclear Resonance Spectroscopy

Nuclear magnetic resonance (NMR) hydrogen spectroscopy usually uses the chemical shift, integral value, peak area, and coupling constant information to infer the specific position of special functional groups on the carbon skeleton and to determine their structure. During the experiment, a small amount of OANND was first dissolved in CDCl_3_, sampled with a syringe and placed in a nuclear magnetic resonance tube, and tested with a nuclear magnetic resonance instrument. The chemical shift change that occurred referred to the internal standard substance tetramethylsilane (TMS).

#### 2.2.3. Evaluation of Blocking Performance

Under the reservoir simulation conditions of 85 °C, a 1038 mD sand pack plugging test was conducted with a backpressure value of 4 MPa and a system injection volume of 0.3 PV to evaluate the plugging performance of the OANND system.

The blocking efficiency can be calculated as follows:

(1) $$\eta =\frac{∆P_{2}-∆P_{1}}{∆P_{2}}\times 100\%$$

where

η represents the blocking efficiency, %.
Δ*P*_1_ represents the stabilized differential pressure value during the first injection of CO_2_, kPa.
Δ*P*_2_ represents the breakthrough pressure difference value during the second injection of CO_2_, kPa.

## 3. Results and Discussion

### 3.1. Infrared and Nuclear Magnetic Analyses

#### 3.1.1. Infrared Spectral Characterization

**Figure 2:**
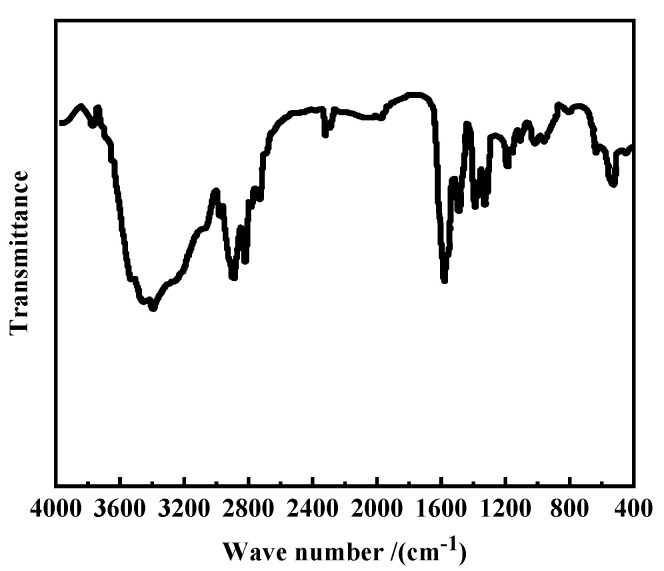
FT-IR spectrum of OANND.

The molecular structure of the material synthesized in our study was characterized using the Fourier transform infrared spectroscopy method, and the results are presented in Figure 2.

As can be observed in Figure 2, the N-H stretching vibration absorption peak of amide is evident at 3410.1 cm^−1^; the stretching vibration absorption peaks of methyl (-CH_3_) and methylene (-CH_2_) are evident at 2898.7 cm^−1^ and 2813.8 cm^−1^, respectively, and the stretching vibration absorption peak of amide carbonyl [29] (C=O) occurs at 1575.3 cm^−1^. The peaks in the IR spectrum reveal the presence of amide groups in the synthesized OANND product, thus indicating that the carboxyl and amino groups reacted to produce the target product, OANND, an oleic acid amidopropyl tertiary amine.

#### 3.1.2. NMR Spectral Analysis

**Figure 3:**
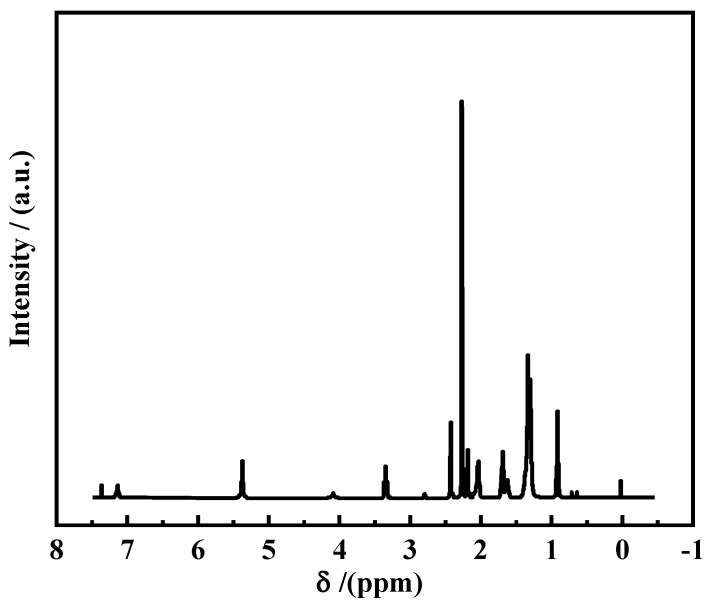
1H-NMR spectrum of OANND.

The functional group composition was further characterized with the nuclear magnetic resonance hydrogen spectroscopy method, and the results are presented in Figure 3.

As can be observed in Figure 3, following the analysis of the ^1^H-NMR (400 MHz, CDCl_3_) mapping results, the peak position at 0.87 corresponds to alkyl-CH_3_, the peak position at 1.30 corresponds to alkyl-CH_2_, the peak position at 1.66 corresponds to C=O in β-CH_2_, the peak position at 2.01 corresponds to C=C in -CH_2_, and the peak position at 2.15 corresponds to C=O in α-CH_2_. The peak at 2.24 corresponds to the N substitution in -CH_3_, peaks at 2.41 and 3.30 correspond to the N substitution in -CH_2_, the peak at 5.33 corresponds to CH=CH, and the peak at 7.11 corresponds to NH [30,31]. The peaks presented above are consistent with the structural formula of the copolymer oleic acid amidopropyl tertiary amine OANND, which suggests the successful production of the target product.

### 3.2. Analysis of Reversible Responsive Behavior

**Figure 4:**
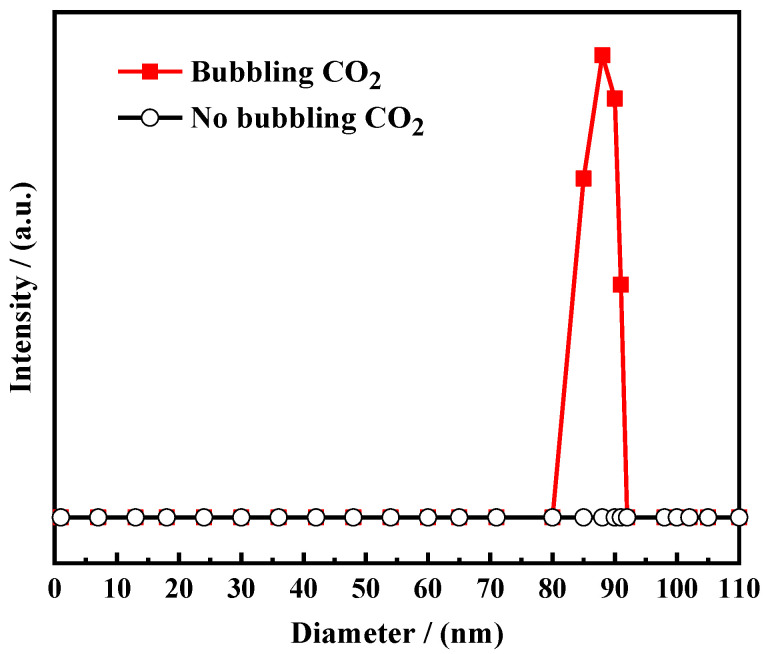
Average size changes in micelles after introduction of CO_2_.

The prepared OANND solution was added to the simulated water plug, and the average size of the particles in the aqueous solution was tested after introducing CO_2_ to the solution for 5 min; the average particle size of its aqueous solution was measured using the dynamic light-scattering technique. Additionally, the results for the average sizes of the particles in the aqueous solution prior and subsequent to the injection of CO_2_ are presented in Figure 4.

As can be observed in Figure 4, the average size of the particles in the aqueous solution of the water plug with the addition of OANND is close to 0 when CO_2_ is not injected into the channel; that is, the average size of the particles in the water plug solution with the addition of OANND is smaller, indicating that OANND is a small-molecule particle. The average size of the micelles following the introduction of CO_2_ for 5 min is 88 nm, and the particle size is relatively similar, which indicates that the system formed stable micelles in a relatively short period of time [32]. This result shows the rapid response of the system after being exposed to CO_2_, ultimately forming a high-strength gel. This occurred because surfactant molecules self-assembled into micelles in the solution through interactions between hydrophobic and hydrophilic groups. The experimental results show that the aggregation morphology of the OANND solution in response to the stimulation of CO_2_ exhibits a particular micelle size and CO_2_ fast-response properties.

**Figure 5:**
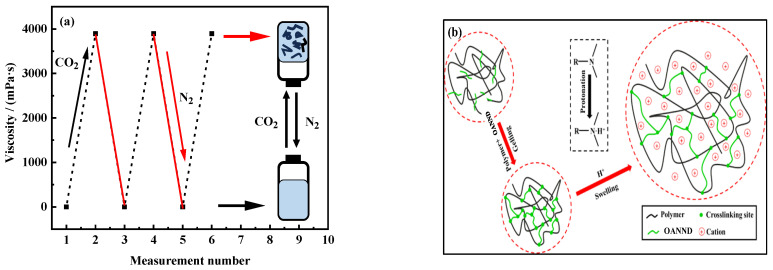
The viscosity (**a**) and mechanism (**b**) of the 100 mM OANND solution in the gas-bubbling cycle.

In the study of CO_2_ intelligent viscoelastic fluids, the reversibility of the polymer system’s viscosity properties under stimulation conditions was also considered an important characteristic of whether the system possessed switching properties. We used 100 mM of the AONND aqueous solution, into which tow gas was alternately passed, to prove the viscosity cycling reversibility potential of the AONND solution system. The relevant data are presented in Figure 5a. Additionally, the relevant mechanism diagram is presented in Figure 5b.

As can be observed in Figure 5a, when CO_2_ is added to the solution, the OANND in the aqueous solution is protonated into an H^+^ cation, and H^+^ combines with organic salt counterion SPTS under an electrostatic condition; the initial spherical micelles in the system transform into flexible micelles, which intertwine with each other to form a dynamic network structure, thus forming a viscoelastic fluid. After the introduction of N_2_ into the system, the deprotonation of H^+^ occurs, the micelles become spherical, and viscosity is reduced. Meanwhile, this high-low viscosity transformation process can be repeated three times, and the highest viscosity value can be maintained at about 4895 mPa·s, indicating that it is a viscosity-reversible system that has a good reusability characteristic. The explanation for the relevant mechanisms involved in this process is presented in Figure 5b.

**Figure 6:**
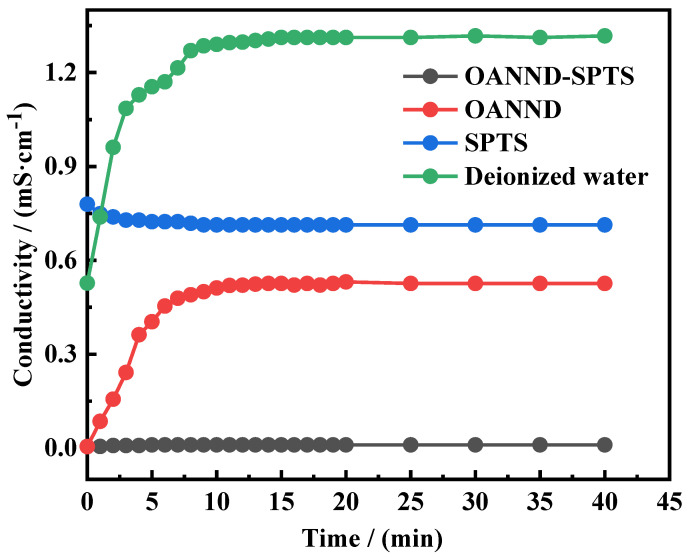
Conductivity curves of 50 mM OANND-SPTS, OANND, and SPTS solutions, and 50 mL of deionized water at 25 °C.

Sodium p-toluenesulfonate (SPTS) was selected as the counterion of the organic salt used in the study, and the OANND-SPTS, OANND, and SPTS solutions, as well as 50 mL of deionized water with a concentration of 50 mM, were prepared at a 1:1 ratio at room temperature. Additionally, we injected CO_2_ into the solution at a rate of 0.1 L/min, and the conductivity changes that occurred in each solution during the ventilation process are presented in Figure 6 in the form of a graph.

Based on the results presented in Figure 6, it can be concluded that the conductivity level of the OANND-SPTS and OANND solutions increased rapidly to the maximum value and reached equilibrium after CO_2_ was introduced into the four solutions. Additionally, the conductivity of the SPTS aqueous solution basically remained unchanged at about 0.71 mS·cm^−1^, while the conductivity change induced by the introduction of CO_2_ into the DI water was minor, which is presented as a straight line at around 0.01 mS∙cm^−1^, thus presenting a significant change in the electrical conductivity.

This result is due to the protonation that occurs under the influence of CO_2_, which generates positively charged H^+^ ions, thus increasing the number of charged ions in the system, which is also supported by the fact that the OANND-SPTS and OANND conductivity curves present the same trend. The SPTS conductivity curve, which remains unchanged, indicates that it is not a CO_2_—responsive polymer, which is due to the fact that the pKa value of the sulfonic acid group carried by SPTS is low, and the carbonic acid created by CO_2_ in water is too weak, so SPTS always exists in the form of sulfonate ions.

### 3.3. The Response of CO_2_ to Polymer Rheology Analysis

**Figure 7:**
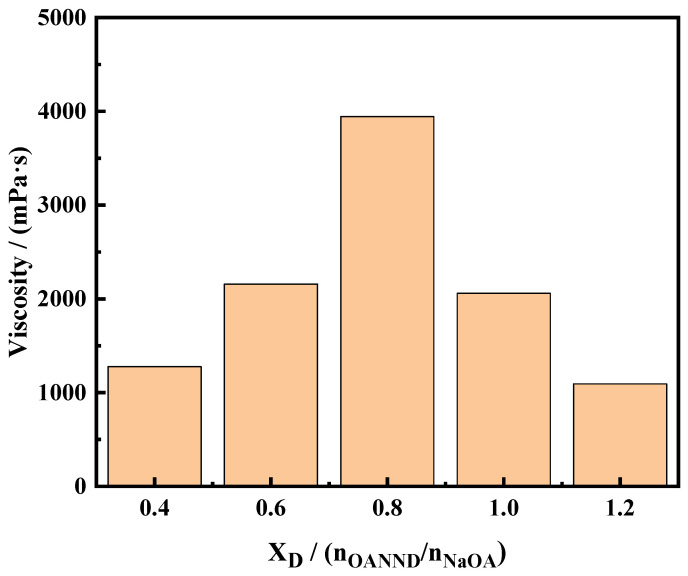
Effect of OANND-to-NaOA molar ratios on viscosity of NaOA solution after bubbling with CO_2_ at 25 °C.

The sodium oleate solution (NaOA), the main agent used in the experiment, was fixed at a value of 70 mM, and the OANND surfactant was added to the solution to achieve a ratio of n_OANND_/n_NaOA_ of 0.4–1.2. The solutions were then evenly mixed under magnetic stirring conditions, and CO_2_ was then introduced to form a uniform and transparent viscoelastic system. The viscosity measurements of each system are presented in Figure 7.

As can be observed in Figure 7, the viscosity of the OANND system is greatly influenced by the molar ratio of additives to the main agent in the system. When CO_2_ is introduced, OANND is protonated to form an OANND-H^+^ cation, and with the gradual increase in X_D_, the active ingredients in the solution increase and more OANND-H^+^ is produced, which can shield part of the negative charge of the main agent (OA^−^), reduce the electrostatic repulsion between the OA^−^ molecules, and increase the hydrophobic chain length of surfactants. Moreover, the more active ingredients present in the solution, the more favorable this is to the intertwining behavior of micelles, thus increasing the viscosity of the system. At the same time, the short-chain cosurfactant OANND also interlinks with negatively charged OA^−^ to encourage the growth of micelles.

However, this electrostatic attraction is saturated at an X_D_ value equal to 0.8. Once X_D_ exceeds 0.8, the excess H^+^ may not only lead to the formation of wormlike micelle branches, but can also provide H^+^ to the system to promote the formation of precipitated HOA; the structure of the entangled wormlike micelle aggregates will be destroyed and, at the same time, the viscosity of the system will be significantly reduced.

**Figure 8:**
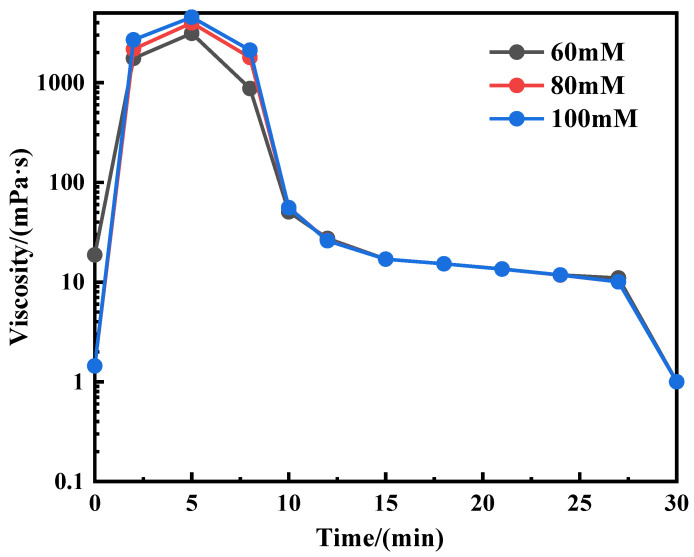
Viscosity change in the process of bubbling CO_2_.

In order to further examine the viscosity behavior of the aqueous solution of the OANND system in the process of introducing CO_2_ into the solution, 60, 80, and 100 mM concentrations of the OANND system (X_D_ = 0.8) solution were prepared at 25 °C, and we continuously introduced CO_2_ into the solution for 0.5 h. By measuring the viscosity of the system, the specific relationship between the duration of CO_2_ exposure and viscosity was determined, and the results are presented in Figure 8.

As can be observed in Figure 8, in the first 5 min of CO_2_ being passed through the solution, the viscosity begins to significantly change and continues to increase. This occurs because the reaction of CO_2_ and water in the solution generates carbonic acid during the ventilation process, and OANND is generated during the process of protonation to become a cation to the main agent of NaOA. OANND is protonated to become a cation during this process, and the induction and binding ability of NaOA to the main agent are enhanced, which causes the viscosity of the system to continuously increase and achieve the highest value when CO_2_ is passed through the solution for 5 min.

**Figure 9:**
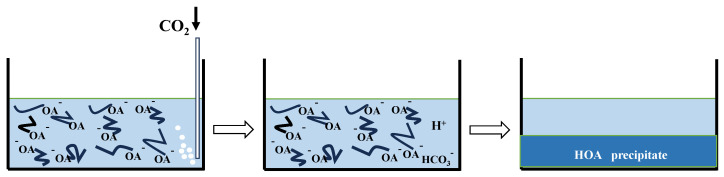
Reaction process of CO_2_ and active NaOA.

However, the viscosity of the OANND system continuously decreases with the continuation of CO_2_ injection, until the viscosity reaches the lowest value. This occurs because of the special characteristics of the main agent, NaOA. When an excessive amount of CO_2_ is added to the solution, the solution produces a large number of H^+^ cations, and it is easy to combine H^+^ cations with OA^−^ to precipitate HOA. Therefore, the active ingredient in the solution to participate in the formation of OA^−^ micelles is greatly reduced, which leads to a reduction in micelles. The viscosity of the system is also reduced, and the specific reaction process is presented in Figure 9.

**Figure 10:**
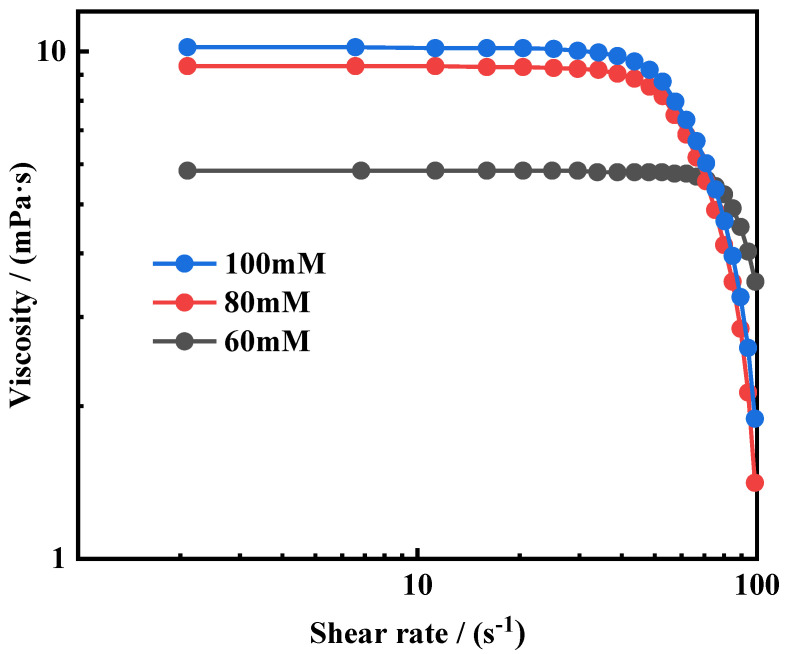
Effect of concentration on shear viscosity.

OANND system solutions at concentrations of 60–100 mM (X_D_ = 0.8) were obtained at room temperature and CO_2_ was passed through them until a viscoelastic system was formed (pH = 9.56 ± 0.1); rheological measurements were performed using a HAAKE RS-6000 rheometer. We performed measurements using a conical plate system (diameter: 60 mm; angle: 1°). Firstly, with a fixed angular frequency of 1 Hz, three different concentrations of OANND CO_2_ (X_D_ = 0.8) sample systems were subjected to the shear stress scanning method to determine linear viscoelastic zones. The viscosity behavior changes in the three samples at different shear rates were measured, and the results are presented in Figure 10.

As shown in Figure 10, all three samples exhibit high-viscosity plateau phenomena at low shear rates and shear-thinning behaviors of non-Newtonian fluids at high shear rates, indicating that a large number of micelle structures formed in the system. Furthermore, as the concentration of the sample increases, the viscosity platform value also increases. This is because in high-concentration systems, many micelles are formed, leading to the further entanglement of the micelle structures in the system, resulting in a higher viscosity value.

**Figure 11:**
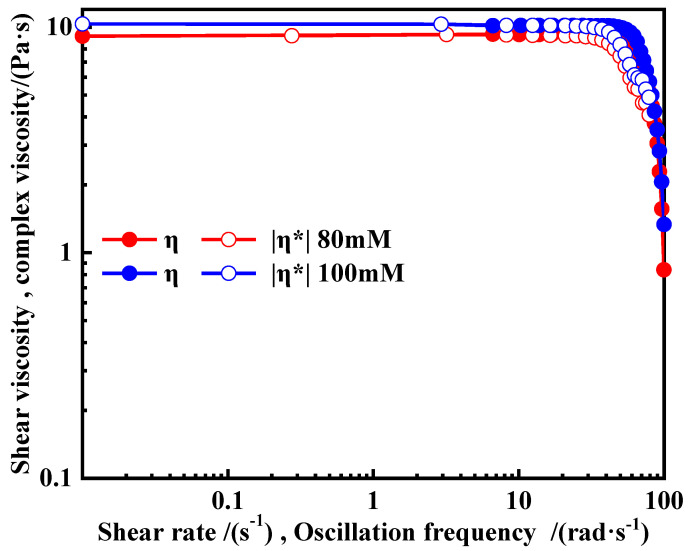
The shear viscosity, η, and complex viscosity (|η*|) values for the OANND-CO_2_ system.

In some of the studies on micelles, the shear viscosity, η, and the complex viscosity, |η*|, the values are equal or similar to the corresponding shear rates and oscillation frequencies for surfactant solutions that form micelles and network structures, a process that is referred to as the Cox-Merz rule [33]. The Cox-Merz rule was applied to 80 and 100 mM values, as well as the OANND-CO_2_ system, and the results are presented in Figure 11 in the form of a graph. The shear and composite viscosities of the two systems change as a result of both the shear rate and oscillation frequency, and the trends of the viscosities remain roughly the same to the same order of magnitude, which is direct evidence of the formation of micelles in the two systems.

### 3.4. Sealing Performance Analysis

The plugging performance of the CO_2_—sensitive gel system was studied and analyzed using a plugging capacity test device of the gel system, and compared with the plugging performance of commercial CO_2_—sensitive precipitation and resin plugging systems. As shown in Table 2, even if the concentration of a gel solution polymer is 1.0–2.0 wt%, the strength of the formed gel is still similar. Additionally, the gel system with a polymer concentration of 1.0 wt% with better injectability results was selected for the blocking capacity experiments. The length of the sand-filled model was 60 cm and the diameter was 2.5 cm.

**Figure 12:**
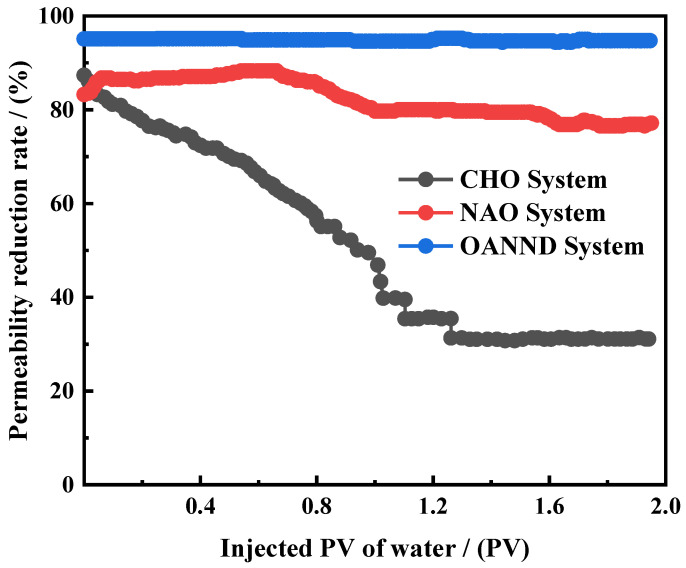
Variations in the shutdown capacity of diverse CO_2_—sensitive systems during water flooding at 85 °C.

The experimental conditions and results determined for the sealing capacity are presented in Table 2 and Figure 12. It can be concluded that the OANND-CO_2_ gel system based on the OANND system can reduce the permeability of the sand-filled model to 95.1% of the initial permeability value at 85 °C. This indicates that this CO_2_—sensitive gel system can effectively seal potential leakage pathways or gas-flushing channels with porous media characteristics and reduce the seepage capacity of fluids in leakage pathways or gas-flushing channels. In the experiment, the permeability of the sand-filled model did not change significantly with the addition of water, and the final permeability value remained at 94.7%.

As can be observed in Table 3, the OANND-CO_2_-sensitive gel system presents better blocking effect and scour resistance results than other commercial CO_2_-sensitive blocking systems used in the field, such as precipitated NAO and water-soluble resin-based CHO blocking systems. When injecting simulated formation water with a value of up to 2PV, the permeability reduction rate of a sodium aluminate solution-based plugging system used in a simulated potential leakage channel is lower than 83%; whereas, due to the brittleness of cured resin, the permeability reduction rate of a water-soluble resin-based CHO plugging system used in a simulated leakage channel is lower than 31% when injecting simulated formation water with a PV value up to 2. In addition, the OANND-CO_2_-sensitive gel system still has a 93.1% permeability reduction rate at 90 °C and with a 2 PV value and displays some temperature resistance.

The results presented in Table 3 clearly indicate that at a temperature of 80 °C, the injection PV of 0.3 of the OANND-CO_2_-sensitive gel system significantly reduced the permeability of the low-permeability sand-filled model by 97–99%. Even at 90 °C, it was observed that with a simulated water mineralization value of 200,000 mg∙L^−1^ and an injected pore volume of 3, the CO_2_-sensitive gel-based plugging system was still capable of reducing the permeability of the high-permeability sand-filled model by over 90% compared to its initial value.

The data suggest that the system maintains a reliable plugging performance in both high-mineralization and -temperature environments. During the second CO_2_ injection round performed to enhance the gel-forming environment, it was observed that the permeability reduction rate increased with an increase in the injection volume, surpassing a value of 80%. This result further indicates that the gel maintains a certain plugging effectiveness result, even under fluid flow conditions.

### 3.5. Double-Tube Oil Repulsion Experiment and Action Mechanism Analysis

**Figure 13:**
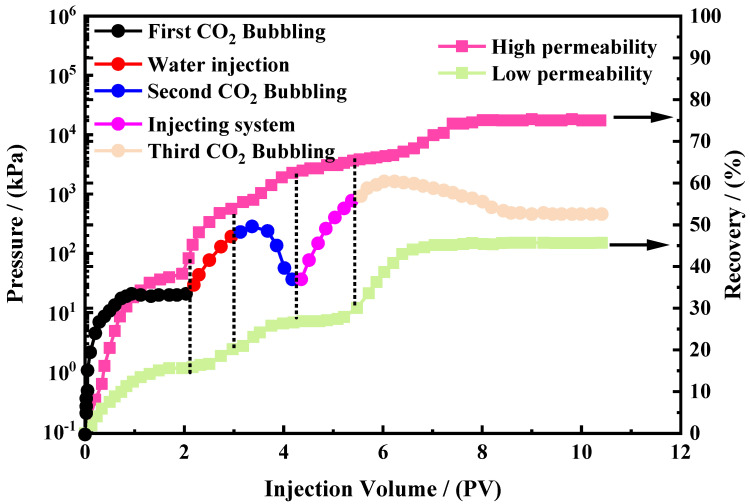
Pressure and recovery rate during experimental process.

We connected two sand-filling pipes in parallel positions, one high-permeability (marked as a high pipe) and one low-permeability (marked as a low pipe) sand-filling pipe, with a permeability ratio of 10:1. We then characterized the uniform permeability of the simulated reservoirs by the corresponding changes occurring in the high- and low-pipe scenarios. The corresponding changes were used to reflect and evaluate the enhanced oil recovery performance of the advanced CO_2_-responsive gel system as an effective flow-control agent. The changes in pressure and recovery values during the five stages of the entire oil recovery process are reflected in Figure 13.

In the first stage, we adopted the first type of CO_2_ bubbling oil displacement technology. By injecting CO_2_ through the inlet, the pressure rapidly increased and gradually stabilized at 33.4 kPa. During this process, CO_2_ preferentially entered the high-porosity and high-connectivity throat region, displacing crude oil. In contrast, the recovery rate of the low pipe was only 15.8%, while the recovery rate of the high pipe was as high as 42.0%. This is because there are many pores in high pipes, and the pore throats have good connectivity.

In the second stage, we used alternating water and gas flooding measures to mitigate gas channeling. When all of the sand packs only produced water, we performed a water drive. During this stage, the increase in pressure prompted the sand packs to discharge a certain amount of oil. The recovery rate of the high pipes reached 53.8%, while the recovery rate of the low pipes was only 20.1%. This indicates that water flows more easily through high pipes.

In the third stage, we conducted a second carbon dioxide drive after the water drive. During this stage, the pressure began to increase to a maximum value. Most of the water entered the high-pressure area, resulting in gas blockage, which delayed the gas breakthrough phenomenon. CO_2_ then entered the low-pressure area, replacing the oil, resulting in an increase in the recovery efficiency of 7.0%. Then, the pressure change caused the gas to re-enter the high pipes; gas re-entered the high-pressure area, resulting in a limited control of gas breakthrough.

In the fourth stage, the intelligent-responsive gel system of water-CO_2_ alternative injection was used to block the low pipes, resulting in low flow resistance, with the pressure first increasing and then decreasing, and a gas channeling control effect. In addition, this affects the pore throat and effectively displaces oil [34].

**Figure 14:**
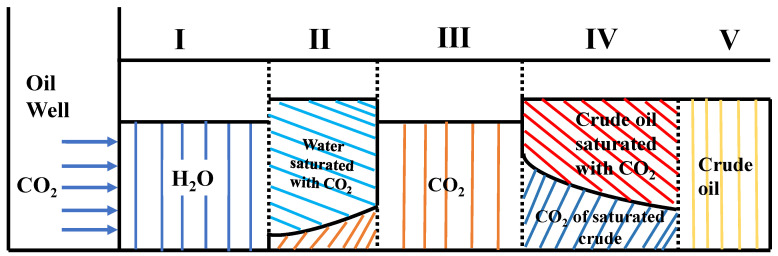
Saturation profile of CO_2_-water alternating gas-bubbling cycle.

The final stage involved deep CO_2_ oil displacement. This required a deeper level of oil displacement from low-level pipes after blocking. Following CO_2_ injection, the water-CO_2_ alternative drive intelligent-responsive gel system formed a high-strength gel, whose viscosity and strength rapidly increased, further plugging the high pipes, and the pressure continued to increase until the gel was broken. At this point, the cumulative recovery rate of the high pipes remained unaltered. The cumulative recovery rate of the low pipes increased by 15.3%. This result shows that the intelligent-responsive gel system of water-CO_2_ alternative flooding selectively blocks the high-penetration region, permitting CO_2_ to access the low pipes for further oil displacement. The recovery rates of both types of filled sand pipes are stable, with a cumulative recovery rate of 75.0% for high pipes and 45.6% for low pipes. This shows that the water-CO_2_ alternative flooding intelligent-responsive gel system can selectively drive oil and control migration rates, which selectively block the high-penetration region, cause the gas to enter the low-penetration region instead of the crude oil, and enhance the crude oil recovery and availability rates. The specific reaction mechanism is presented in Figure 14.

## 4. Conclusions

In order to improve the effect of the CO_2_ and water alternating displacement process, a new type of CO_2_-responsive polymerization technique was developed with specific features.

1. Oleic acid (OA) and N,N-dimethyl-1,3-propanediamine (NND) were synthesized in one step using the fatty acid method to synthesize intelligent-responsive polymers for water-CO_2_ alternating drive controlled gas scavenging activity. The peak positions of the IR spectra demonstrate the presence of characteristic amide groups in the synthesized OANND product. The peak positions of the NMR spectra are consistent with the structural formula of the copolymer oleic acid amidopropyl tertiary amine, OANND, confirming the generation of the target product.

2. The system was viscosity-reversible and demonstrated good reusability. The average size of its micelles reached approximately 88 nm after passing CO_2_ through them for 5 min, and the particle size was relatively homogeneous, indicating that stable micelles were rapidly formed so that the system was protonated by the OANND after exposure to CO_2_, forming a high-strength gel.

3. The intelligent-responsive gel system of water-CO_2_ alternative flooding selectively influenced oil and control migration rates, which selectively blocked the high-penetration region, caused the gas to enter the low-penetration region instead of the crude oil, and enhanced the crude oil recovery and availability rates. The CO_2_-sensitive gel system could effectively seal potential leakage pathways or gas-flushing channels with porous media characteristics and reduce the seepage capacity of fluids in leakage pathways or gas-flushing channels. In addition, the OANND-CO_2_-sensitive gel system retained a 93.1% permeability reduction at 90 °C and with a value of 2PV, which presented a certain level of temperature resistance.

## Figures and Tables

**Table 1:** Experimental instruments and equipment.

Instrument	Model Number	Manufacturer	Conditions
Conductivity meter	DDS-11A	Jintan Melting Instrument Manufacturing Co., Ltd. Changzhou, China	Normal temperature and pressure
Thermostatic heating magnetic stirrer	CJJ78-1	Jintan Dadi Automation Instrument Factory, Changzhou, China	Normal temperature and pressure
Rotary evaporator	R206B	Shanghai Shensheng Technology Co., Ltd. Shanghai, China	Normal temperature and pressure
Rotary viscometer	Bookfield DV-III	Ametec Blechfeld China Co., Ltd. Shanghai, China	Normal temperature and pressure
Rheometer	HAAKE RS-600	Thermo Fisher Scientific, Waltham, MA, USA	Normal temperature and pressure
Circulating water vacuum pump	SHZ-Ⅲ	Gongyi Yuhua Instrument Co., Ltd. Zhengzhou, China	Normal temperature and pressure
Electronic balance	ALC-104	Mettler Toledo Instruments (Shanghai) Co., Ltd. Shanghai, China	Normal temperature and pressure

**Table 2:** Gel performance with different polymer concentrations.

Polymer Concentration/wt%	N_2_ Environment		CO_2_ Environment	
	Gelation Time/h	Strength Code	Gelation Time/h	Strength Code
0.5	>24 h	A	1.81	F-B
1.0	>24 h	A	2.03	H
1.5	>24 h	A	2.25	H
2.0	>24 h	A	2.49	H

**Table 3:** Comparison of shutoff capacity between advanced intelligent-responsive polymers for CO_2_-responsive polymer gel system with concentration of 1 wt% and conventional CO_2_-sensitive gel system.

System	Temperature/°C	Salinity of Formation Water/mg∙L^−1^	Sand Pack		Injected Water Volume/PV	Maximum Permeability Reduction Rate/%	Final Permeability Reduction Rate/%
			Pore Volume/mL	Initial Permeability/×10^−3^ μm^2^			
OANND	90	200,000	110	1698.5	3	94.5	93.1
	80	20,000	100	19.4	2	96.5	94.2
	70	20,000	100	59.6	2	98.7	97.9
	70	20,000	109	120.2	2	97.9	97.2
NAO	80	20,000	98	31.6	2	89.4	82.8
CHO	80	20,000	105	26.1	2	87.5	30.4

## Data Availability

No new data was created or analyzed in this study.
